# Associations of physical activity with cognitive function and daily physical function among Chinese individuals with heart disease: A cross-sectional study

**DOI:** 10.3389/fpubh.2022.917390

**Published:** 2022-11-22

**Authors:** Xiaosheng Dong, Xiangren Yi, Ningxin Jia, Meng Ding, Yanan Zhou, Caijun Tian

**Affiliations:** ^1^Department of Sport and Health, School of Physical Education, Shandong University, Jinan, China; ^2^College of Physical Education, Shandong Normal University, Jinan, China; ^3^Department of Traditional Chinese Medicine Classics, University of Traditional Chinese Medicine, Jinan, China

**Keywords:** heart disease, physical activity (PA), daily physical function, cognitive function, activities of daily living (ADL)

## Abstract

**Background:**

To investigate the associations between different dimensions of physical activity (PA), cognitive function, and daily physical function in Chinese individuals with heart disease.

**Materials and methods:**

This study included 2,792 individuals from the China Health and Retirement Longitudinal Study conducted in 2015. Physical activity (PA) was divided into vigorous PA (VPA), moderate PA (MPA), and light PA (LPA). Linear and logistic regression models were established to assess the associations among the indicators.

**Results:**

Compared with taking no PA, MPA, and VPA at a frequency of 6–7 d/w had lower risks of impaired daily physical function (OR = 0.47, 95% CI: 0.25, 0.91; OR = 0.57, 95% CI: 0.37, 0.88) and higher cognitive function scores (β = 1.22, 95% CI: 0.42, 2.03; β = 1.08, 95% CI: 0.43, 1.73), while VPA at 3–5 d/w had lower cognitive function scores (β = −1.96, 95% CI: −3.51, −0.40). Light PA (LPA) with a duration of 30–119 min/d had a lower risk of impaired daily physical function (OR = 0.59, 95% CI: 0.36, 0.97). Moderate PA (MPA) and VPA of 30–119 min/d had higher cognitive function scores (β = 1.43, 95% CI: 0.49, 2.37; β = 1.30, 95% CI: −0.56, 2.06). The 1,800–2,999 METs had the lowest risks of impaired daily physical function and the highest cognitive function scores (OR = 0.18, 95% CI: 0.04, 0.75; β = 2.94, 95% CI: 1.67, 4.21).

**Conclusion:**

Moderate PA (MPA) and LPA with a frequency of 6–7 d/w and a duration of 30–119 min/d, and PA in 1,800–2,999 MET min/week were most closely related to better cognitive and daily physical function, while VPA (3–5 d/w; ≥300 min/w) may be related to low cognition, but high-quality research is necessary to prove causality.

**Trial registration:**

IRB00001052-11015.

## Introduction

Heart disease is a major non-communicable disease worldwide and it has been the leading cause of death for the past two decades (1). Its prevalence exhibits a continual increase and it imposes a heavy burden on society, which has become a major public health problem (2). Ischemic heart disease mortality increased by 155.4% between 1990 and 2017 in China, resulting in a severe economic burden (3).

Current studies have shown that patients with heart disease are at risk for deterioration of cognitive abilities, such as memory loss (4). Patients with an increased cardiac burden typically exhibit reduced hippocampal, cortical gray matter, and total brain volumes and exacerbated cognitive decline (5, 6); it has been observed that the greater the cardiac burden is, the faster the rate of decline of episodic and working memory (7, 8). Additionally, related studies have shown that cognitive impairment among patients with heart disease is negatively correlated with their function of activities of daily living (ADL) (9). Damage to the structure of their physiological system and a deterioration of their cognitive function can induce severe symptoms in patients, reduce their quality of life (10), and may also limit their ability to perform ADLs (11). Heart disease–induced cognitive decline has been found to be more detrimental than physical failure to patients over time (12). The progression of their disease course accelerates their cognitive decline as well as a deterioration of their ability to perform ADLs, while cognitive decline also accelerates their course of disability (5).

Previous studies demonstrated that levels of physical activity (PA) are strongly associated with the level of health in patients with heart disease and can improve their psychological, cognitive, and social functioning (13, 14). Physical activity has also been found to improve attention, processing speed, executive function, memory, and structural brain integrity (15). However, there is no large representative sample study about the associations between PA and cognitive function or daily physical function in patients with heart disease. Therefore, the aim of this study was to investigate the associations between different dimensions (intensity, frequency, duration, volume, and metabolic equivalent) of PA and the cognitive function and daily physical functioning of Chinese individuals with heart disease.

## Materials and methods

### Study population

The data used in this study are from the China Health and Retirement Longitudinal Study (CHARLS) conducted in 2015. The CHARLS is a nationally representative longitudinal cohort study. Through stratified cluster sampling, subjects were selected from 28 provinces in China, and a follow-up survey was conducted every 2 years. China Health and Retirement Longitudinal Study (CHARLS) is the authoritative micro survey data on the health status of the elderly in China. The Ethics Committee of Peking University Health Science Center has approved CHARLS. The ethical approval number is IRB00001052-11015. All participants provided written informed consent for participation.

**Figure 1:**
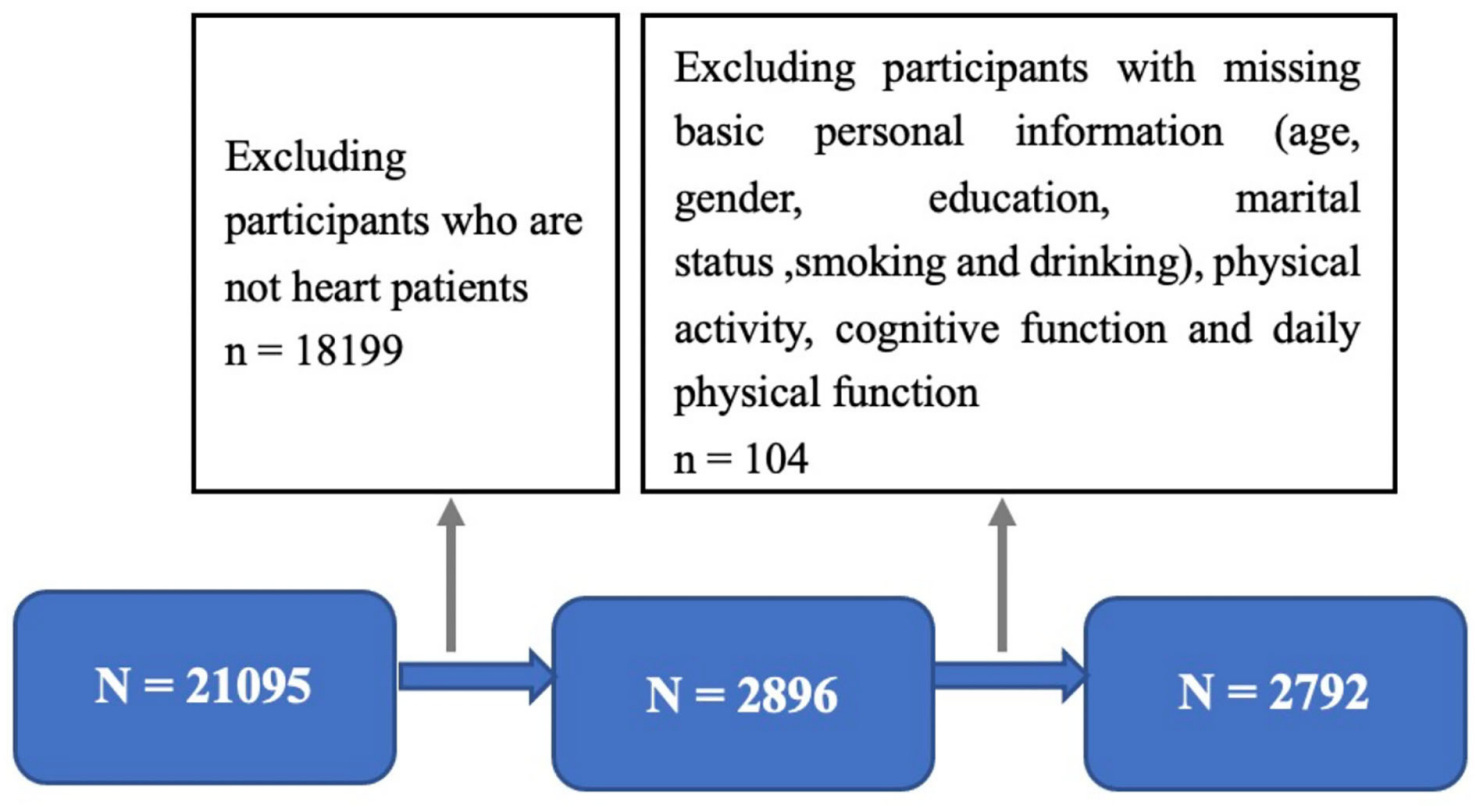
Participants selected from survey in 2015.

A total of 21,096 participants were investigated in 2015. Participants included in the study had complete data, such as age, height, weight, gender, educational level, marital status, smoking, drinking, PA record, and diagnosis of heart disease. Samples with missing data, outliers, or logical errors were excluded. In addition, the subjects of this study were patients with heart disease, and the vast majority of the CHARLS participants were excluded from this study because they did not have heart disease. Finally, 2,792 patients with heart disease were included in the final statistical analysis. A detail procedure of selection is shown in Figure 1.

### Heart disease measurements

Patients with heart disease were defined through the question “Have you been diagnosed with heart disease by a doctor?” and “Are you now taking any of the following treatments because of your heart disease and its complications? Taking traditional Chinese medicine, taking Western medicine, taking other treatments than taking medicine.” We considered that the individual had heart disease when the response was “yes” to either one or both of the questions.

### Assessment of PA

Each subject reported their amount of weekly physical activity, stratified into vigorous physical activity (VPA) (which makes you short of breath, such as carrying heavy objects, digging, farming, aerobic exercise, fast cycling, and cycling to load goods); moderate physical activity (MPA) (causing you to breathe faster than usual, such as carrying light things, riding a bicycle at a regular speed, mopping the floor, playing Taijiquan, and sprinting); and light physical activity (LPA) (walking from one place to another when working or at home and other walks for leisure, sports, exercise, or entertainment). The subject was asked, “Do you usually do VPA/MPA/LPA for at least 10 min every week?” If “No” was selected, VPA/MPA/LPA was deemed not to be performed. If the answer was “Yes”, they were further asked, “How many days per week do you usually do VPA/MPA/LPA?” and “How long do you spend on VPA/MPA/LPA each time?” The frequency of PA ranged from 0 to 7 d/w and was separated into four levels: no activity (0 d/w), 1–2, 3–5, and 6–7 d/w. The duration of PA was categorized into five levels: no activity, 10–29, 30–119, 120–239, and ≥240 min/d. Considering that the average of each duration is used in the questionnaire instead of the specific duration (16), we calculated the total volume of VPA/MPA/LPA in a week by multiplying the frequency and duration of VPA/MPA/LPA. In addition, referring to WHO guidelines (17), the total amount of VPA was divided into four levels: no activity, 10–74, 75–299, and ≥300 min/w, while the total time per week of MPA/LPA was divided into four levels: no activity, 10–149, 150–299, and ≥300 min/w.

According to previous studies, 1 MET refers to the amount of oxygen consumed during sitting (3.5 mL O_2_/kg/min), and the intensity of high-intensity exercise can be expressed as 8 METs, moderate-intensity exercise can be expressed as 4 METs, and walking can be expressed as 3.3 METs. The number of minutes of high-intensity activity, medium-intensity activity, and walking per week is calculated by multiplying the METs (i.e., the multiple of the resting energy consumption). Total physical activity (MET) was calculated as the sum of the scores for VPA + MPA + LPA (16). According to the guidelines of the American College of Sports Medicine (ACSM) (18), the minimum level of total physical activity beneficial to health is defined as 600 MET min/week. Therefore, these categories are multiples of CDC and ACSM physical activity recommendations. MET min/week is divided into nine categories: 0 to < 600, 600–1,199, 1,200–1,799, 1,800–2,999, 3,000–5,999, 6,000–8,999, 9,000–11,999, ≥12,000).

### Assessment of daily physical function

Daily physical function was measured by the Activities of Daily Living scale and the Instrumental Activities of Daily Living (IADL) scale (19). Each option on the two scales was divided into four levels: “No difficulty = 0”, “Difficult but still can be completed = 1”, “Difficult but need help = 2”, and “Cannot complete = 3”, and the comprehensive score range was 0–43. According to previous studies, this article defines an ADL/IADL comprehensive score ≥11 as a loss of physical activity function and < 11 as no loss of function (19). Using Cronbach's α (α = 0.887), the reliability of the questionnaire was established.

### Assessment of cognitive function

Cognitive function was evaluated through three kinds of tests: the Telephone Interview of Cognitive Status (TICS) (orientation and attention), word recall (episodic memory), and figure drawing (visuospatial ability) (20). Telephone Interview of Cognitive Status (TICS) (orientation and attention) (21): the subjects were asked to answer the current year, month, date, day of week, and season and additionally were asked to calculate 100 minus 7 and then 5 consecutive reductions. Then, the scores for the correct questions were summed. Word recall (episodic memory) (22, 23): subjects were asked to remember and immediately recall as many words as possible in arbitrary order after reading a page displaying 10 nouns in Chinese (immediate recall). After 4–10 min, the subjects were asked to recall as many original words as possible (delayed recall). The episodic memory score was the average score of immediate recall and delayed recall. Figure drawing (visuospatial ability) (22): the subjects were shown a picture of two overlapping pentagons and asked to draw a similar picture. If they succeeded, they got 1 point and if not, they got 0 point. The cognitive function score was the sum of the above three parts, and the higher the score, the better the cognitive function.

### Assessment of covariables

The covariates selected from the participants in this study include age (continuous variable), height (continuous variable), weight (continuous variable), gender (male, female), education level (junior high school and below, high school or vocational school, junior college, and above), marital status (married or unmarried, separated or divorced or widowed, never married), smoking status (never, ever, and now), and drinking status (never, before, and now).

### Data analysis

The categorical variables of this study are expressed by numbers (*n*) and percentages (%), and the continuous variables are expressed by means and standard deviations (mean ± standard deviation). Linear regression or binary logistic regression was used to analyze the relationships among the different dimensions of PA, metabolic equivalent, daily physical activity, and cognitive level. For linear regression data β, the coefficient and 95% confidence interval (CI) are expressed, and binary logistic regression is expressed by odds ratio (OR) and 95% confidence interval (CI). All models were adjusted for the potential confounding covariates of age, sex, education, marital status, alcohol consumption, smoking, and BMI. Data were analyzed using the software IBM SPSS Statistics for Windows (Statistics 23, IBM Corporation, New York, USA). A *p*-value < 0.05 was considered statistically significant in all analyses.

## Results

### Characteristics of the participants

Our study included 2,792 participants, which consisted of 1,090 males (38.7%) and 1,712 females (61.3%). Among them, 2,311 patients (82.9%) were married or living with a partner, 2,209 patients (79.1%) had a junior high school or below education level, 1,735 patients (59.7%) did not smoke, and 1,915 patients (68.6%) did not drink alcohol. The basic characteristics of the participants are shown in Table 1.

**Table 1:** Basic characteristics of participants.

**Characteristic**	**Overall sample (*****N*** = **2,792)**	
	**Mean**	**SD**
Age, year	65.26	9.85
BMI, kg/m^2^	24.94	4.04
**Sex (*****n*** **+** **%)**		
Male	1,080	38.7
Female	1,712	61.3
**Marital status (*****n*** **+** **%)**		
Married or partnered	2,311	82.8
Separated, divorced, or widowed	464	16.6
Never married	17	0.6
**Educational status (*****n*** **+** **%)**		
Junior high school or below	2,209	79.1
Senior high school or vocational school	385	13.8
College or above	198	7.1
**Smoking (*****n*** **+** **%)**		
Never	1,735	62.1
Former	480	17.2
Current	572	20.5
**Drinking (*****n*** **+** **%)**		
Never	1,915	68.6
Former	146	5.2
Current	731	26.2

*SD*, standard deviation; *BMI*, body mass index.

### Frequency of PA

The relationship between the frequency of PA and daily physical function is shown in Table 2. Compared with individuals taking no PA, individuals taking MPA (OR = 0.47, 95% CI: 0.25, 0.91) and VPA (OR = 0.57, 95% CI: 0.37, 0.88) with a frequency of 6–7 d/w had lower risks of impaired daily physical function. Furthermore, as the relationship between frequency of PA and cognitive function shows (Table 2), individuals with MPA 1–2 d/w (β = 1.51, 95% CI: 0.02, 3.00) and 6–7 d/w (β = 1.22, 95% CI: 0.42, 2.03) all had higher cognitive function scores, individuals with LPA 3–5 d/w (β = 2.06, 95% CI: 0.75, 3.36) and 6–7 d/w (β = 1.08, 95% CI: 0.43, 1.73) all had higher cognitive function scores, while individuals taking VPA 3–5 d/w had lower scores of cognitive function (β = −1.96, 95% CI: −3.51, −0.40).

**Table 2:** Associations between physical activity and daily physical activity ability and cognitive function of patients with heart disease.

**Variables**	**Daily physical function**		**Cognitive function**	
	**OR**	**95% CI**	**β**	**95% CI**
**Frequency**				
VPA				
No activity	1.00		1.00	
1–2 d/w	N/A	N/A	−0.53	−2.26, 1.20
3–5 d/w	1.48	0.43,5.11	−1.96	−3.51, −0.40^*^
6–7 d/w	1.09	0.41,2.90	−0.92	−2.03, 0.19
MPA				
No activity	1.00		1.00	
1–2 d/w	0.38	0.09,1.62	1.51	0.02, 3.00^*^
3–5 d/w	0.25	0.06,1.08	0.76	−0.50, 2.01
6–7 d/w	0.47	0.25,0.91 ^*^	1.22	0.42, 2.03^**^
LPA				
No activity	1.00		1.00	
1–2 d/w	1.59	0.53,4.79	0.80	−1.18, 2.77
3–5 d/w	0.52	0.19,1.47	2.06	0.75, 3.36^**^
6–7 d/w	0.57	0.37,0.88 ^*^	1.08	0.43, 1.73^**^
**Duration**				
VPA				
No activity	1.00		1.00	
10–29 min/d	N/A	N/A	0.58	−3.05, 4.21
30–119 min/d	0.79	0.18,3.43	−1.06	−2.72, 0.59
120–239 min/d	0.98	0.23,4.27	−1.29	−2.86, 0.29
≥240 min/d	0.97	0.32,2.99	−0.99	−2.22, 0.24
MPA				
No activity	1.00		1.00	
10–29 min/d	0.51	0.15,1.67	1.78	0.34, 3.21^*^
30–119 min/d	0.64	0.32,1.27	1.43	0.49, 2.37^**^
120–239 min/d	0.30	0.09,1.01	0.98	−0.17, 2.13
≥240 min/d	N/A	N/A	0.67	−0.59, 1.93
LPA				
No activity	1.00		1.00	
10–29 min/d	0.47	0.20,1.11	1.18	−0.08, 2.29^*^
30–119 min/d	0.59	0.36,0.97 ^*^	1.30	0.56, 2.04^**^
120–239 min/d	0.58	0.27,1.25	0.83	−0.16, 1.82
≥240 min/d	1.32	0.55,3.18	0.75	−0.56, 2.06
**Volume**				
VPA				
No activity	1.00		1.00	
10–74 min/w	N/A	N/A	−0.66	−5.60, 4.28
75–299 min/w	N/A	N/A	−0.71	−2.74, 1.33
≥300 min/w	1.06	0.45, 2.48	−1.18	−2.14, −0.23^*^
MPA				
No activity	1.00		1.00	
10–149 min/w	0.57	0.22, 1.49	1.67	0.46, 2.87^**^
150–299 min/w	0.95	0.22, 4.15	2.02	−0.18, 4.06
≥300 min/w	0.35	0.17, 0.72 ^**^	0.89	0.09, 1.68^*^
LPA				
No activity	1.00		1.00	
10–149 min/w	0.64	0.31, 1.33	1.25	0.19, 2.30^*^
150–299 min/w	0.27	0.04, 2.04	1.24	−0.69, 3.16
≥300 min/w	0.59	0.38, 0.92 ^*^	1.26	0.59, 1.93^**^

*OR*, odds ratio; β, Regression coefficients; *CI*, confidence interval; *PA*, physical activity; *VPA*, vigorous physical activity; *MPA*, moderate physical activity; *LPA*, light physical activity; Model was adjusted for age, sex, educational status, marital status, drinking, smoking, and BMI; N/A denoted that no applicable value was observed;

* *p*<*0.05;*

** *p*<*0.01*.

### Duration of PA

Table 2 shows the relationship between the duration of PA and daily physical function. Compared with individuals taking no PA, there was no significant risk of impaired daily physical function between MPA and VPA of any duration, whereas only individuals taking LPA for a duration of 30–119 min/d had a lower risk of impaired daily physical function (OR = 0.59, 95% CI: 0.36, 0.97). Furthermore, as the relationship between the duration of PA and cognitive function shows (Table 2), compared with individuals taking no PA, individuals with MPA 10–29 min/d (β = 1.78, 95% CI: 0.34, 3.21) and 30–119 min/d (β = 1.43, 95% CI: 0.49, 2.37) all had higher cognitive function scores, and individuals with LPA 10–29 min/d (β = 1.18, 95% CI: −0.08, 2.29) and 30–119 min/d (β = 1.30, 95% CI: −0.56, 2.06) all had higher cognitive function scores.

### Volume of PA

Table 2 shows the relationship between the volume of PA and daily physical function. Compared with individuals taking no PA, individuals taking MPA (OR = 0.35, 95% CI: 0.17, 0.72) and LPA (OR = 0.59, 95% CI: 0.38, 0.92) with a volume of ≥300 min/w had lower risks of impaired daily physical function, whereas there was no significant between VPA of any volume and risks of impaired daily physical function. Furthermore, the relationship between the volume of PA and cognitive function showed (Table 2) lower scores of cognitive functions in individuals taking no PA and ≥300 min/w (β = −1.18, 95% CI: −2.14, −0.23). A volume of 10–149 min/w, of ≥300 min/w in MPA (β = 1.67, 95% CI: 0.46, 2.87; β = 0.89, 95% CI: 0.09, 1.68) and LPA (β = 1.25, 95% CI: 0.19, 2.30; β = 1.26, 95% CI: 0.59, 1.93) all had higher cognitive function scores.

### METs

The relationships among METs, daily physical function, and cognitive function are shown in Table 3. Compared with individuals taking no PA, individuals taking 1,800 to < 2,999 METs, 3,000 to < 5,999 METs, and 6,000 to < 8,999 METs had lower risks of impaired daily physical function and higher cognitive function scores. Furthermore, individuals with 1,800–2,999 METs had the lowest risks of impaired daily physical function (OR = 0.18, 95% CI: 0.04, 0.75) and the highest scores for cognitive function (β = 2.94, 95% CI: 1.67, 4.21).

**Table 3:** Associations between METs and the daily physical activity ability and cognitive function of patients with heart disease.

**Variables**	**Daily physical function**		**Cognitive function**	
	**OR**	**95% CI**	**β**	**95% CI**
**Mets**				
0 to < 600				
600 to < 1,199	0.31	0.07, 1.31	1.28	−0.49, 3.05
1,200 to < 1,799	0.60	0.34, 1.06	1.29	0.317, 2.26 ^**^
1,800 to < 2,999	0.18	0.04, 0.75 ^*^	2.94	1.67, 4.21 ^**^
3,000 to < 5,999	0.29	0.14, 0.60 ^**^	1.23	0.40, 2.06 ^**^
6,000 to < 8,999	0.24	0.08, 0.77 ^*^	1.32	0.21, 2.43 ^*^
9,000 to < 11,999	0.29	0.07, 1.19	0.71	−0.75, 2.17
≥12,000	0.42	0.17, 1.04	0.98	−0.10, 2.05

*OR*, odds ratio; β, regression coefficients; *CI*, confidence interval; Model was adjusted for age, sex, educational status, marital status, drinking, smoking, and BMI;

* *p*<*0.05;*

** *p*<*0.01*.

## Discussion

The results of this study have shown that high frequency (3–5 d/w) and high volume (≥300 min/w) VPA in patients with heart disease was correlated with poor cognitive function, MPA and LPA with a frequency of 6–7 d/w and a duration of 30–119 min/d, and PA in 1,800-2,999 MET min/week were most closely related to better cognitive function and better daily physical function.

With regard to VPA, we found that high-intensity exercise (3–5 d/w; ≥300 min/w) was associated with worse cognition in patients with heart disease and had nothing to do with physical function. As far as we know, previous studies (24) also found that high-intensity exercise may reduce the cognition of patients with heart disease. In addition,cognitive function is associated with various processes related to specific regions of the brain (25). Competition between different brain regions for limited metabolic resources would occur during VPA, and if more metabolic resources are allocated to the motor and sensory cortices during VPA, this would be at the expense of metabolic resources supplied to the prefrontal cortex (26). Vigorous PA (VPA) impairs metabolism in the prefrontal cortex of the brain in patients with heart disease and ultimately reduces cognitive performance (27). Cerebral blood flow is increased by the activation of local neurons to meet the metabolic demand (28); during exercise, cerebral blood flow is regulated by a complex interplay among neuronal activity and metabolism, blood pressure, sympathetic nervous system activity, the partial pressures of oxygen and carbon dioxide, and the cardiac output (29). Cerebral blood flow during exercise depends on the intensity of the exercise, and during maximal exercise, cerebral blood flow decreases progressively, mainly as a result of hyperventilation, suggesting that the cerebral metabolic demand may be unmet during high-intensity exercise (29), which may be one of the reasons why the results of the present study showed worse cognition in patients with cardiac disease and VPA (3–5 d/w; ≥300 min/w). In addition, it has been shown that patients have impaired cardiac function and that the myocardium may not be able to pump a sufficient blood supply; thus, VPA intervention does not necessarily improve mobility in patients with coronary heart disease or heart failure (30), which is consistent with the result in the current study that VPA has no association with the ability of daily physical function in patients with heart disease.

With regard to MPA and LPA, we found that patients with heart disease who engaged in MPA and LPA every week had better cognitive (MPA: 1–2, 6–7 d/w, 10–119 min/d; LPA: 3–7 ssd/w, 10–119 min/d, 10–149 min/w, ≥300 min/w) and daily physical functions (MPA: 6–7 d/w, ≥300 min/w; LPA: 6–7 d/w, 30–119 min/d, ≥300 min/w), and those with ≥300 min/w had the best cognitive and daily physical functions. The current study confirmed cardiorespiratory fitness as a health indicator strongly associated with mortality, functional health, and cognitive decline (31). The relationship between exercise intensity and cognition was assumed to be an inverted U-shape (32), and MPA and LPA are easier to achieve and more acceptable to patients. Australian and British guidelines recommend that patients engage in light- to moderate-intensity aerobic exercise for cardiac rehabilitation (33). Furthermore, autonomic regulation ability and neurotransmitter function were positively correlated with the frequency of PA (34), and improvements in neurotransmitter function and autonomic regulatory capacity were most marked in patients with heart disease who underwent 6 days of light- to moderate-intensity aerobic exercise per week. Studies have shown that frequent exercise (≥6 times/week) in patients with heart disease is associated with improved cardiopulmonary exercise capacity and psychosocial function, with significant improvements in mobility (35), which is consistent with the findings from this study that 6–7 days per week of MPA and LPA have benefits for both cognition and daily physical function in patients with heart disease. In addition, previous studies confirmed that a sustained 45 min of exercise not only improves attention, memory, and visuospatial abilities in patients with heart disease but also increases serum brain-derived neurotrophic factor (BDNF) levels and cerebral blood flow in the middle cerebral artery (36) MPA of 75 min per week had the best effect on cognition (37), which is compatible with the results in the present study showed that 30–119 min/d of MPA and LPA have the most beneficial effects on cognition and daily physical function. However, the present study found that the duration of MPA was not associated with daily physical function. The controversial effects of PA on daily physical function have been reported in previous relevant studies, which may be related to the intensity of exercise (38). Additional high-quality studies are still needed. Relevant studies have shown that MPA and LPA may play a protective role in the ability to perform daily physical function, and MPA and LPA have a significant favorable effect on physical performance indicators of daily physical function, with MPA having the greatest effect (39). In addition, MPA and LPA contribute to cognition, whereas vigorous, very vigorous, and maximal intensity PA confers no benefit to cognition (40). The apparent measurable decline in cognition found after VPA may be explained by the fact that prolonged VPA can lead to dehydration and exhaustion of energy stores, which can lead to cognitive decline (41), which is consistent with the result in this study that MPA and LPA ≥300 min/w conferred the most benefit on cognition and daily physical function in patients with heart disease.

With regard to MET, our results showed that patients with cardiac disease and 1,800–8,999 METs had better cognition and daily physical function than patients with insufficient exercise, and patients with 1,800–2,999 METs min/week had the best cognition and daily physical function. Previous studies have shown that patients with cardiac disease and less than 3,000 METs/week of PA had larger gains in daily physical function and diminishing returns above 3,000 METs/week of PA (42). Physical activity (PA) delays the decline in cognitive function, and MPA (140 × 4 × 4 = 2,240 METs) may have the most beneficial effects (43), which were all consistent with the results of the present study. Therefore, limiting physical activity in patients with heart disease within reasonable metabolic equivalents is likely to result in better cognitive function and better improvements in daily physical function.

We used a large representative sample from China in this study. However, this study is a cross-sectional study, which cannot accurately explain or establish any cause-and-effect relationship, and there was no detailed differentiation of PA. Then, the results of this study may be unstable due to insufficient sample size and other reasons, which will affect the accuracy of the conclusion of this study. In addition, in this study, samples with incomplete data were excluded, which may have a certain impact on this study. Finally, this study determined whether the patient suffered from heart disease through a self-reported doctor diagnosis, without considering the influence of risk factors, such as systemic arterial hypertension, major structural diseases, or surgical correction of diseases. Additional high-quality studies are needed to explore these issues.

This study revealed a relationship between different dimensions (intensity, frequency, duration, volume, and metabolic equivalent) of PA and cognitive function and daily physical function among Chinese individuals with heart disease. After adjustment for potential confounders, VPA (3–5 d/w; ≥300 min/w) was associated with lower cognitive function in the total sample but not with daily physical function. Moderate PA (MPA) and LPA were beneficial to the cognitive function (MPA: 1–2, 6–7 d/w, 10–119 min/d; LPA: 3–7 d/w, 10–119, 10–149 min/w, ≥300 min/w) and daily physical functions (MPA: 6–7 d/w, ≥300 min/w; LPA: 6–7 d/w, 30–119 min/d, ≥300 min/w) of Chinese individuals with heart disease, and a duration of 30–119 min/d had the most benefit, whereas there was no significant correlation between total physical function and MPA of any duration. A total of 1,800–8,999 METs were beneficial to cognitive function and daily physical activity, and 1,800–2,999 METs had the most benefit. These findings provide additional evidence about the associations between PA and cognitive function and daily physical function among Chinese individuals with heart disease. In view of the limitations of this article, it is necessary to carry out longitudinal and randomized controlled trials to verify causality in future. In addition, it is necessary to explore the benefits of resistance training and aerobic exercise further among patients with heart disease to provide information to guide exercise recommendations for this population.

## Data availability statement

The datasets presented in this study can be found in online repositories. The names of the repository/repositories and accession number(s) can be found in the article.

## Ethics statement

The studies involving human participants were reviewed and approved by the Ethics Committee of Peking University Health Science Center, the ethical approval number was IRB00001052-11015. The patients/participants provided their written informed consent to participate in this study.

## Author contributions

CT was involved in the conceptualization and investigation. NJ, YZ, MD, and XD conducted the analysis and interpretation of the data. XD drafted the initial manuscript. XY substantively revised the work. All authors read and approved the final manuscript.

## Funding

This study is supported by The Exercises Promote Health Theory and Practice Innovation Team of Shandong Normal University in China (No. 112/14001).

## Conflict of interest

The authors declare that the research was conducted in the absence of any commercial or financial relationships that could be construed as a potential conflict of interest.

## Publisher's note

All claims expressed in this article are solely those of the authors and do not necessarily represent those of their affiliated organizations, or those of the publisher, the editors and the reviewers. Any product that may be evaluated in this article, or claim that may be made by its manufacturer, is not guaranteed or endorsed by the publisher.
